# Development and Validation of a Self-Administered Online Hearing Test

**DOI:** 10.1177/23312165251317923

**Published:** 2025-03-18

**Authors:** Charlotte Vercammen, Olaf Strelcyk

**Affiliations:** 1 87724 Sonova AG, Research & Development, Stäfa, Switzerland; 2 Manchester Centre for Audiology and Deafness, School of Health Sciences, 12203 Faculty of Biology, Medicine and Health, University of Manchester, Manchester, UK; 3 Department of Neurosciences, 596886 Research Group Experimental Oto-Rhino-Laryngology, University of Leuven (KU Leuven), Leuven, Belgium; 4 Sonova U.S. Corporate Services, LLC, Cincinnati, OH, USA; 5 Department of Otolaryngology and Communicative Disorders, 5170 University of Louisville, Louisville, KY, USA

**Keywords:** hearing loss, hearing screening, audiogram, telehealth, computational audiology

## Abstract

We describe the development and validation of a self-administered online hearing test, which screens for hearing loss and provides an estimated audiogram. The hearing test computes test results from age, self-reported hearing abilities, and self-assessed pure-tone thresholds. It relies on regression, Bayesian and binary classification, leveraging probabilistic effects of age as well as interfrequency and interaural relationships in audiograms. The test was devised based on development data, collected prospectively in an online experiment from a purposive convenience sample of 251 adult American, Australian, Canadian, and Swiss participants, 58% of whom had hearing loss. Later, we externally validated the hearing test. Validation data were collected prospectively from a representative sample of 156 adult Belgian participants, 15% of whom had hearing loss. Participants completed the hearing test and audiometric assessments at home. The results for the primary screening outcome showed that the hearing test screened for mild hearing losses with a sensitivity of 0.83 [95%-confidence interval (CI): 0.65, 0.96], specificity of 0.94 [CI: 0.89, 0.98], positive predictive value of 0.70 [CI: 0.57, 0.87], and negative predictive value of 0.97 [CI: 0.94, 0.99]. Results for the secondary audiogram estimation outcome showed mean differences between estimated and gold standard hearing thresholds ranging from 2.1 to 12.4 dB, with an average standard deviation of the differences of 14.8 dB. In conclusion, the hearing test performed comparably to state-of-the-art hearing screeners. This test, therefore, is a validated alternative to existing screening tools, and, additionally, it provides an estimated audiogram.

## Introduction

One in five individuals worldwide is affected by some degree of hearing loss (HL). By 2050, one in four will be affected—an estimated 2.5 billion individuals globally (World Health Organization, 2021b). Yet, more than 80% of individuals who could benefit from HL management remain undiagnosed or untreated (Orji et al., 2020). The global economic burden resulting from unaddressed HL is valued at more than 980 billion US dollars per year (World Health Organization, 2021b). This burden is primarily attributed to years lived with disability, lost productivity, and non-hearing-related healthcare costs (McDaid et al., 2021). HL in midlife is also associated with dementia, but is considered one of its strongest “potentially modifiable” risk factors, along with dyslipidemia (Livingston et al., 2024).

Hearing screening and provision of amplification are cost-effective ways to manage sensorineural HL (World Health Organization, 2017). Early access to interventions might reduce the associated global burden. Therefore, clinical practice guidelines recommend adult hearing screening from the age of 50 years onwards—at every health care encounter (Tsai Do et al., 2024) or at proposed intervals of one to five years depending on risk factors (American Speech-Language-Hearing Association, 2006; World Health Organization, 2021a). For adults below the age of 50 years, the American Speech-Language-Hearing Association (2006) recommends hearing screening at intervals of 10 years. Hearing testing (and care) could be offered remotely to overcome geographic restrictions, reduce the cost of care, and unburden clinicians (Wilson et al., 2017). Pre-requisites for this are validated hearing assessment tools that are suitable for remote and/or self-administered use. In 2021, Irace et al. reviewed mobile applications that were publicly available for self-evaluation of hearing abilities and could only connect 16% (seven out of 44) to a validation study. In 2022, Almufarrij et al. provided an overview of commercial instruments intended for remote assessment of hearing abilities available as smartphone or web-based applications. Of the 187 instruments considered, 12% had been formally evaluated and discussed in peer-reviewed publications. A mere 7% were deemed reliable and accurate.

The digits-in-noise test (for a review, see Van den Borre et al., 2021), is likely the most well-known and accepted self-administered hearing test available today. It is endorsed by the World Health Organization and available through their hearWHO mobile application (World Health Organization, n.d.). The digits-in-noise test relies on the suprathreshold presentation of digit triplets in the presence of background noise. The masking effect of ambient background noise is typically smaller than that of the noise presented as part of the test, making it more suitable for testing in home environments. And, due to the low complexity of the digit triplet stimuli, the influence of cognitive abilities on test scores is expected to be small (Jansen et al., 2013). One limitation of the digits-in-noise tests (and other tests evaluating speech intelligibility) is that they are language-specific. Consequently, a separate test must be developed for every language (De Sousa et al., 2021). This can hamper standardization and normalization. Also, test results are provided in terms of percentage-correct scores, speech reception thresholds, or pass/fail results (when obtained scores are better/poorer than a priori defined cutoffs). Hearing care, however—including the provision of amplification—typically requires at least an audiometric evaluation (Roeser et al., 2007), that is, knowledge of an individual's hearing thresholds determined as the softest pure tones they can detect across frequency.

Pure-tones-in-quiet testing is problematic when self-administered or performed remotely, because stimuli are presented at threshold level and in quiet. This makes testing susceptible to acoustic uncertainties, such as those resulting from the use of uncontrolled or uncalibrated equipment and the presence of ambient noise in the test environment (De Sousa et al., 2021). Untrained individuals, further, may introduce errors in usage. Nevertheless, pure-tone audiograms are the gold standard for diagnosing hearing loss (Roeser et al., 2007) and can be interpreted by clinicians as well as trained non-specialist personnel. Audiograms are language-independent and are applicable worldwide. The latter is attractive from the perspective of mapping and monitoring hearing loss globally—especially if the audiogram could be estimated using a simple, short, online hearing test. The COVID-19 pandemic further intensified the need for a remote hearing test that would not only reliably screen for HL but also estimate audiograms. When an in-person visit to a clinic was not feasible, an estimated audiogram could, for example, be used for pre-fitting customization of hearing aids, such as preliminary gain conditioning and vent selection, prior to delivering the devices to the patient. Once the patient received the hearing aids, in-situ audiometry should be performed (Van Eeckhoutte et al., 2024; Vercammen, 2020), for instance during a remote support session. Similar procedures may support hearing rehabilitation in low- and middle-income communities, where innovative models of service delivery are needed (Dillard et al., 2024; World Health Organization, 2023). In view of all of this, we aimed to develop and validate a self-administered online hearing test that was designed for home use. The primary aim of the hearing test was to reliably screen for HL. The secondary aim was to estimate a full bilateral audiogram.

For the development of the hearing test, which took place during the COVID-19 pandemic, we purposively recruited 251 participants with available gold standard clinical audiograms. We aimed at including an equal number of individuals with and without HL (see Methods section for full details). The following data were collected prospectively in an online study: information about the individual's age decade, self-report multiple-choice responses to questions about hearing abilities (e.g., “*How would you describe your hearing?*”—see Methods section for a full list of questions), and self-measured pure-tones-in-quiet hearing thresholds along with information about the headphones or earphones used for the measurements. Based on these data, we developed a predictive algorithm that computed the envisaged hearing test results. The algorithm, as described in detail in the Methods section, relied on multiple regression (Royston & Sauerbrei, 2008), Bayesian classification (Özdamar et al., 1990), and binary classification (Chicco, 2017; Zweig & Campbell, 1993).

Later, we externally validated the hearing test (see Altman et al., 2009), based on a dataset collected prospectively from a sample of 156 participants, representative of typical users of online hearing tests (see Methods section). The primary aim of the hearing test was to reliably screen for HL. Primary outcome metrics for the validation were sensitivity, specificity, positive, and negative predictive values. We used gold standard clinical audiometry as a reference. The secondary aim of the hearing test was to estimate full bilateral audiograms. Secondary outcome metrics for the validation were the mean differences between estimated and gold standard hearing thresholds, that is, the estimated bias, as well as the standard deviation of the differences. This hearing test was not intended to replace diagnostic testing. We nevertheless have provided detailed descriptions along the lines of the 2015 STARD checklist outlining Standards for Reporting Diagnostic Accuracy (Bossuyt et al., 2015) in the Supplemental material (Table S1). This should allow for easier comparison with other studies as suggested by Almufarrij et al. (2022).

## Methods

### Development Dataset

Development data were collected between June and September of 2020 in Australia, Canada, Switzerland, and the USA via an online web application. The participants constituted a purposive convenience sample (Andrade, 2021) recruited from existing research and clinical databases with the goal of including an equal number of individuals with and without HL. They were considered for inclusion if they were 18 years or older and had undergone clinical audiometric testing within the previous two years. Individuals were contacted via e-mail, mail, or telephone. They were informed about the study and the logistical requirements for taking part in it, i.e., they needed access to a smartphone, tablet, or computer with an internet connection, and a pair of headphones or earphones. Those who were interested in taking part received the study documents via mail or e-mail.

An initial sample of *n* = 254 participants was recruited (Australia: *n* = 47; Canada: *n* = 104; Switzerland: *n* = 46; the USA: *n* = 57). Three participants (Canada: *n* = 1; Switzerland: *n* = 2) were excluded due to incomplete audiometric information. Three participants had missing hearing threshold values at 8 kHz but were included. For all other participants, pure-tone hearing thresholds were available in research or clinical databases for both ears at 0.5, 1, 2, 4, 6, and 8 kHz. The sample was balanced in terms of gender, with 47% male and 44% female participants (9% missing values).

Participants were instructed to access a web application remotely via their own smartphone, tablet, or computer (according to their own preference). Once they accessed the web application, they were asked to respond to seven queries about their age, self-perceived hearing abilities, and type of headphones or earphones they were using for this test (see Table 1). Subsequently, they were instructed to ensure that they were in a quiet environment, set the volume settings of the devices to 50%, and perform self-administered pure-tone threshold measurements at 0.5, 1, 2, 4, and 6 kHz. The threshold testing was done separately for both ears using the following instructions, which were displayed on the screen of the web application: “Press the play button to start the tone. Use +/− buttons to adjust the loudness and find the softest sound you can hear. Then press next.” Instructions were translated to German for participants recruited in Switzerland. Testing started with the presentation of a 0.5-kHz tone in the right ear at a level of –50 dB relative to full scale (FS). Each press of the “+/-” buttons increased/decreased the tone level by 5 dB. The last level that was set before pressing the button “Next” was logged as the measured pure-tone threshold in dB FS. The initial presentation level at each subsequent tone frequency was set to the threshold level of the preceding tone incremented by 15 dB. There were no missing values for data collected through the web application, and no adverse events were reported during data collection.

**Table 1.** Overview of Questions Posed to Participants During Development Data Collection (First Column), Possible Response Options (Second Column), and how Responses Were Coded in the Development of the Hearing Test's Predictive Algorithm (Third Column).

Query	Response options	Numerical coding
Q_1_: Select your age range.	18–29 / 30–39 / 40–49 / 50–59 / 60–69 / 70–79 / 80–89 / 90+	
Q_2_: How would you describe your hearing?	good / not sure / poor	1 /2 /3
Q_3_: Do you find it hard to follow one-on-one conversation or do people seem to mumble?	always / often / sometimes / rarely / never	1 / 2 / 3 / 4 / 5
Q_4_: Do you find it hard to have a conversation on the phone?	always / often / sometimes / rarely / never	1 / 2 / 3 / 4 / 5
Q_5_: Do you find it hard to hear high-pitched sounds like bird song?	always / often / sometimes / rarely / never	1 / 2 / 3 / 4 / 5
Q_6_: Do you find it hard to follow conversations in noisy settings like crowded restaurants?	always / often / sometimes / rarely / never	1 / 2 / 3 / 4 / 5
Q_7_: Select your headphone or earphone style and put them on.	on-ear, cables / in-ear, cables / on-ear, wireless / in-ear, wireless	

The median time between data collection via the web application and last available clinical audiogram was 48 weeks (with an interquartile range of 16 weeks). The type of smartphone, tablet, or computer used by the participants during data collection was automatically identified and registered by the web application. The data was collected in real-world test conditions, with all individuals utilizing their own readily available equipment. Sixty-three percent of the participants used a Windows PC, 16% used an Apple Macintosh computer, 8% used an iPad, 7% used an iPhone, 3% used a Samsung mobile phone, 2% used a Samsung tablet, and the remaining 2% used unrecognized devices. As the type of headphones or earphones could not be automatically identified by the web application, users were asked to indicate the type of transducer they were using before proceeding with the threshold measurements (see Q_7_ in Table 1).

### Hearing Test

The development of the hearing test proceeded from the development dataset and was performed in R (R Core Team, 2020). The hearing test would feature the same interface as the web application that was used to collect the development data, but would be augmented by a predictive algorithm that would compute the test results. As described in the Introduction, the purpose of the hearing test was to serve a dual function, primarily screening for HL, secondarily estimating an individual's full bilateral audiogram. However, the calculation of the screening outcome (pass/fail) was based on the estimated audiogram. Therefore, we will begin by describing the parts of the algorithm that computed the estimated audiogram and then will delineate the subsequent calculation of the screening outcome.

#### Audiogram Estimation

The first step in the predictive algorithm was an approximate conversion of the self-administered pure-tone thresholds from dB FS to dB HL. This was accomplished by performing calibration measurements of the hearing test tones produced by various devices and transducers on a Kemar with anthropometric ears. The conversion to dB HL values then followed a comparison of these measurements with corresponding measurements using Sennheiser HDA200 headphones (ANSI, 2018). Rather than performing conversions for each device and transducer combination, average conversions were applied within categories such as Windows PC with wired transducer, Apple Macintosh computer with wired transducer, iPad with wired transducer, Android tablet with wired transducer, iPhone with wired transducer, Android phone with wired transducer, Windows PC with wireless transducer, Apple Macintosh computer with wireless transducer, and so on. After finishing the development data collection, we decided that future users of the hearing test should set their device volumes at 100% rather than 50%, for sake of simplicity. Therefore, the dB FS threshold values were corrected for the output level differences between the 50% and 100% volume settings for each of the device categories.

One important objective of the predictive algorithm was to yield an audiogram estimate that was more robust to the uncertainties and errors inherent in self-administered pure-tone threshold measurements than methods that would have simply used the measured dB FS thresholds converted to dB HL as the audiogram estimate. Potential measurement uncertainties and errors could, for example, result from environmental noise, uncontrolled playback devices, and incorrectly set device volumes. Therefore, we developed a multivariate regression model that integrated the data from both self-administered threshold measurements and self-reported hearing abilities as a second step in the predictive algorithm. This model used multiple fractional polynomials (Royston & Sauerbrei, 2008), which extend linear regression by applying a power *p* or logarithmic transformation to the predictors. We chose fractional polynomials here to construct a parsimonious and interpretable model that would nevertheless account for non-linear relationships (Royston & Sauerbrei, 2008). The model used the clinical pure-tone thresholds as dependent variables. At each of the tone frequencies from 0.5 to 6 kHz, we tested the following predictors in forward selection: self-administered pure-tone threshold converted to dB HL at the corresponding frequency and responses to queries Q_2_ to Q_7_ (see Table 1). The self-administered pure-tone thresholds were transformed by a 20-dB upwards shift and subsequent division by 100 dB, resulting in the transformed predictor $t_{\;f,e}$ , with *f* and *e* coding frequency and ear, respectively. This was done to ensure positivity and align the scaling of the predictors (Royston & Sauerbrei, 2008). The responses to the queries *Q_n_* were coded as numerals *q_n_* (see Table 1). The aim was to select the three most significant predictors for each frequency. Item Q_1_ about age was not included as a potential predictor since the development dataset was too sparsely sampled in terms of age. The influence of age will be revisited further below. At all frequencies, the first two selected predictors were the self-administered pure-tone threshold at the corresponding frequency and item Q_2_ about general self-perceived hearing ability (*p *< .0001). For low-to-mid-frequencies (0.5 and 1 kHz), item Q_4_ about difficulties with conversations on the telephone was selected as the third predictor (*p *< .01), whereas for higher frequencies (2, 4, and 6 kHz), item Q_5_ about difficulties hearing high-pitched sounds was selected as the third predictor (*p *< .0001). The reduced model predicted medial thresholds $t_{f,e}^{\prime }$ according to the following equation:

(1) $$\begin{array}{rl} t_{f,e}^{\prime }= & \;c_{0,f,e}+c_{1,f,e}\times {t_{f,e}}^{\;p_{1,f,e}}+c_{2,f,e}\times {q_{2}}^{\;p_{2,f,e}}+c_{3,f,e}\times {q_{k}}^{\;p_{3,f,e}}\\ \mathrm{with}\;k(f)= & \{\begin{array}{c} 4,\;\;\mathrm{for}\;f\epsilon \;\{0.5,\;1\;\mathrm{kHz}\}\\ 5,\;\;\mathrm{for}\;f\epsilon \;\{2,\;4,\;6\;\mathrm{kHz}\} \end{array} \end{array}$$

where $c_{n,f,e}$ were real coefficients and $p_{n,f,e}$ were integer powers. Parameterwise jackknife shrinkage factors were applied to the model coefficients to produce more robust estimates in terms of decreased prediction errors (Dunkler et al., 2016; Efron & Tibshirani, 1994; Royston & Sauerbrei, 2008).

In the above, we referred to the thresholds $t_{\;f,e}^{\prime }$ as medial thresholds because they did not represent the final pure-tone hearing threshold estimate. Instead, in a third step the medial thresholds were used as input to a naïve Bayes classifier which also considered the individual's age and produced the final estimated audiogram. It accomplished this by comparing the medial thresholds to a set of bilateral pure-tone audiogram prototypes that were representative of the individual's age decade in the population at large and selecting the most likely audiogram prototype. This most likely audiogram prototype, consisting of left-ear and right-ear pure-tone hearing thresholds as a function of frequency, was the estimated audiogram returned by the predictive algorithm. The procedure was similar to the Bayesian audiogram classification procedure described by Özdamar et al. (1990), albeit it being applied to audiogram estimation rather than classification in the present case and the medial thresholds $t_{\;f,e}^{\prime }$ replacing Özdamar et al.'s test trial response outcomes.

The set of representative audiogram prototypes used by the Bayes classifier was not directly based on an existing dataset, since we considered the available representative datasets to be too small. Instead, we used a resampling approach. First, we derived representative pure-tone average distributions for each age decade (using the mean of left-ear and right-ear four-frequency pure-tone averages across 0.5, 1, 2, and 4 kHz, PTA_4_) from 14,887 bilateral audiograms in the National Health and Nutrition Examination Survey (NHANES) datasets between 1999 and 2016. This constituted a nationally representative sample of the audiograms in the adult U.S. civilian, non-institutionalized population. Next, we merged the NHANES audiograms with a larger dataset of 144,276 bilateral audiograms from a Sonova audiometric database of hearing aid users. Then, for each age decade, we resampled from this merged dataset such that the PTA_4_ distribution of the resampled dataset for a given age decade matched the representative PTA_4_ distribution for that age decade derived from NHANES. Finally, *k*-means clustering was applied to the resampled audiogram data for each age decade. The *k* cluster centers constituted the set { $A_{n}$ } of bilateral audiogram prototypes for each age decade.

Given the medial thresholds $t_{\;f,e}^{\prime }$ and the prototype set { $A_{n}$ } specific to the individual's age, the naïve Bayes classifier derived the estimated bilateral audiogram as the audiometric prototype $A_{\hat{n}}$ that maximized the unnormalized posterior $p(A_{n}|t^{\prime })$ :

(2) $$\begin{array}{rl} \hat{n}= & arg\underset{n}{\max}\;p(A_{n}|\;t^{\prime })\;\;\mathrm{with}\\ p(A_{n}|\;t^{\prime })= & p(A_{n})\times p(t^{\prime }|\;A_{n})\\ = & p(A_{n})\times \underset{\;f,\;e}{\prod }\;p(t_{f,\;e}^{\prime }|\;A_{n}) \end{array}$$

where the informative prior $p(A_{n})$ was given as the relative cluster size, i.e., the number of resampled audiograms in the cluster with cluster center $A_{n}$ divided by the total number of audiograms. The term: $p(t_{f,e}^{\prime }|\;A_{n})$ represented the likelihood of observing a threshold $t_{f,e}^{\prime }$ given the audiogram prototype $A_{n}$ . The process by which the threshold was determined was modeled as a situation in which the individual once heard and once did not hear the tone:

$$\begin{array}{rl} \;p(t_{f,e}^{\prime }|\;A_{n})= & \mathrm{cdf}(t_{f,e}^{\prime },\mathrm{mean}=A_{n,f,e},\mathrm{sd}=\sigma_{f,e})\\ & \times [1-\mathrm{cdf}(t_{f,e}^{\prime },\mathrm{mean}=A_{n,f,e},\mathrm{sd}=\sigma_{f,e})], \end{array}$$

where the psychometric function $\mathrm{cdf}(t_{f,e}^{\prime },\mathrm{mean}=A_{n,f,e},$ $\mathrm{sd}=\sigma_{f,e})$ was the value of the cumulative normal distribution function with mean $A_{n,f,e}$ (the value of $A_{n}$ at frequency *f* in ear *e*) and standard deviation $\sigma_{f,e}$ at $t_{f,e}^{\prime }$ .

We computed predicted performance metrics in terms of the root mean square error, mean difference, and standard deviation of the differences by means of Bootstrap simulation (Efron & Tibshirani, 1994) to account for the over-optimism of the model resulting from the model being fitted and evaluated on the same development dataset. We then selected values for the parameters *k* (number of audiogram prototypes) and $\sigma_{f,e}$ that minimized the predicted root mean square error averaged across frequency with the additional constraint that the predicted mean difference averaged across frequency should be within the range of ±1 dB. This process was repeated for various frequency sets in Equations (1) and (2) of the ten tone threshold measurements included in the development dataset, for example, considering measured thresholds at 0.5, 1, 2, 4, and 6 kHz in both left and right ears, or considering thresholds at 1 kHz in the left ear, 4 kHz in the right ear, and 2 and 6 kHz in both ears, or considering thresholds at 1 kHz in the left ear, 4 kHz in the right ear, and 6 kHz in both ears, etc. If we had taken the measured thresholds $t_{f,e}$ or the medial thresholds $t_{\;f,e}^{\prime }$ as actual threshold estimates, estimation performance would have decreased with decreasing number of tone measurements. However, this was not the case for the full model since the Bayesian backend exploited probabilistic effects of age as well as interfrequency and interaural concordances in human audiograms (Barbour et al., 2019). The predicted root mean square errors, mean differences, and standard deviations of the audiogram estimation errors made by the full model did not worsen when decreasing the set size from ten measurements to four measurements. Therefore, we chose to include four measurements in the final hearing test: 1 and 6 kHz in the left ear, and 2 and 4 kHz in the right ear. Furthermore, we observed that replacing the 0.5- and 8-kHz thresholds of the estimated audiogram by constant next-neighbor extrapolation from 1 to 0.5 kHz and from 6 to 8 kHz increased estimation performance. Therefore, the final hearing test used this extrapolation. The selected value for the parameter *k* was 1,100 prototype audiograms per age decade. The Bootstrap simulation yielded predicted mean differences between estimated and gold standard hearing thresholds ranging from −1.7 to 2.6 dB depending on ear and frequency and a predicted standard deviation of the differences of 15.0 dB averaged across ear and frequencies from 0.5 to 8 kHz.

**Figure 1.**
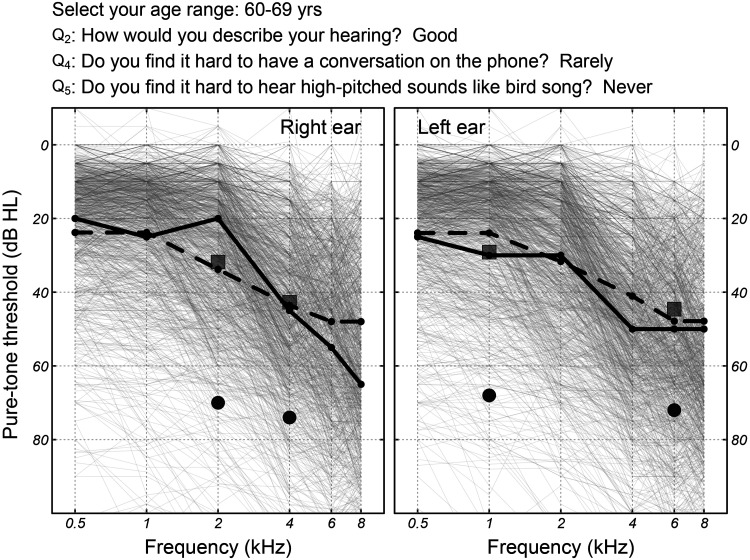
An illustration of the hearing test's audiogram predictive algorithm for a participant in the development dataset. Age range selection and responses to questions Q_2_, Q_4_, and Q_5_ are shown on top of the graph. The solid black curves show the participant's true clinical air-conduction audiogram. Bullets show the four tone thresholds $t^{\prime }\left(f,e\right)$ , self-assessed as part of the online hearing test. Squares show the medial thresholds $t_{\;f,e}^{\prime }$ , i.e., the output of the regression model. Dashed black curves show the estimated audiogram selected by the Bayesian backend from the audiogram prototypes which are visualized as solid gray curves.

Figure 1 illustrates the hearing test's audiogram estimation for a participant in the development dataset. Age range selection and responses to questions Q_2_, Q_4_, and Q_5_ are shown on top of the graph. The solid black curves show the participant's clinical pure-tone audiogram, while the bullets show the four measured tone thresholds *t_f_*_,*e*_ which overshot the audiogram for unknown reasons (possibly due to faulty volume settings, background noise, usage error, etc.). The squares show the medial thresholds $t_{\;f,e}^{\prime }$ . They are shifted upward by the regression model because this participant indicated that they rarely to never experienced hearing difficulties. Finally, the Bayesian backend combined the medial thresholds across frequency and ears producing the estimated audiogram (dashed black curves) by selecting it from the 1,100 prototype audiograms (solid gray curves).

#### Pass/Fail HL Screening Outcome

The final stage of the hearing test algorithm was a binary classifier that used the estimated audiogram described in the previous section as its input. The classifier was construed to give a reliable pass/fail recommendation. It calculated a summary statistic of the estimated hearing thresholds and determined a pass/fail screening outcome if the statistic was better/poorer than a cutoff point. If the individual passed the screener, they would be advised to take the test again in the future. If they failed, they would be advised to contact a clinician for follow-up. As pure-tone audiometry is the gold standard for diagnosing HL (Roeser et al., 2007), the binary classifier was optimized in reference to clinical pure-tone audiometry. For this HL screening application, we chose a mild-HL degree as the referral criterion. We defined this mild-HL degree as a clinical five-frequency pure-tone average (PTA_5_) across 0.5, 1, 2, 4, and 6 kHz of 35.0 dB HL in at least one ear. This value was derived from the standard N2 audiogram, representing mild HL (Bisgaard et al., 2010). We used a five-frequency pure-tone average that included 6 kHz to accentuate high-frequency HLs in comparison with low-frequency HLs. This was done in order to enhance the likelihood of detecting mild high-frequency losses early, thereby ensuring timely access to interventions. The WHO classification of HL degree uses the PTA_4_ instead of the PTA_5_ (Humes, 2018). Taking the mild-HL standard N2 audiogram as a basis, this would yield a corresponding PTA_4_ criterion value of 31.3 dB HL.

**Figure 2.**
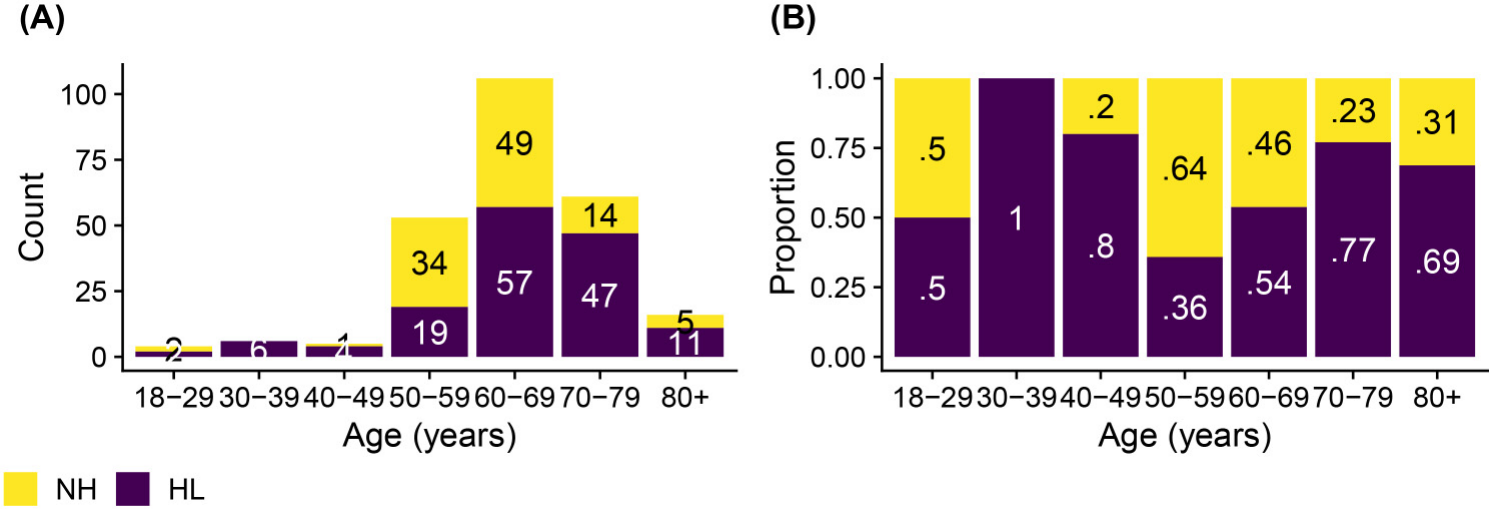
The number (panel A) and proportion (panel B) of participants in the development dataset (*n* = 251) as a function of their age decade. Hearing status (NH versus HL) was determined per the mild-HL criterion of a PTA_5_ greater than 35 dB HL in at least one ear. In panel B, each bar adds up to 100%.

The development dataset (*n* = 251) was relatively balanced (see Figure 2). It included 146 (58%) participants who met the (PTA_5_) mild-HL criterion and 105 (42%) participants who did not. For the sake of brevity, the latter group will be referred to as NH in the following. A balanced dataset was imperative to optimize the binary classifier so as not to bias the classifier toward the largest category (Chicco, 2017). The binary classifier was optimized using receiver operating characteristics (ROCs; Chicco, 2017; Zweig & Campbell, 1993). Analyses were performed in R using the *pROC*-package (Robin et al., 2011). Ninety-five percent confidence intervals (CIs) for the primary outcome metrics were determined based on non-parametric stratified bootstrap resampling (10,000 bootstrap samples) and using Delong's method for the area under the ROC curve (AUC; Robin et al., 2011).

**Figure 3.**
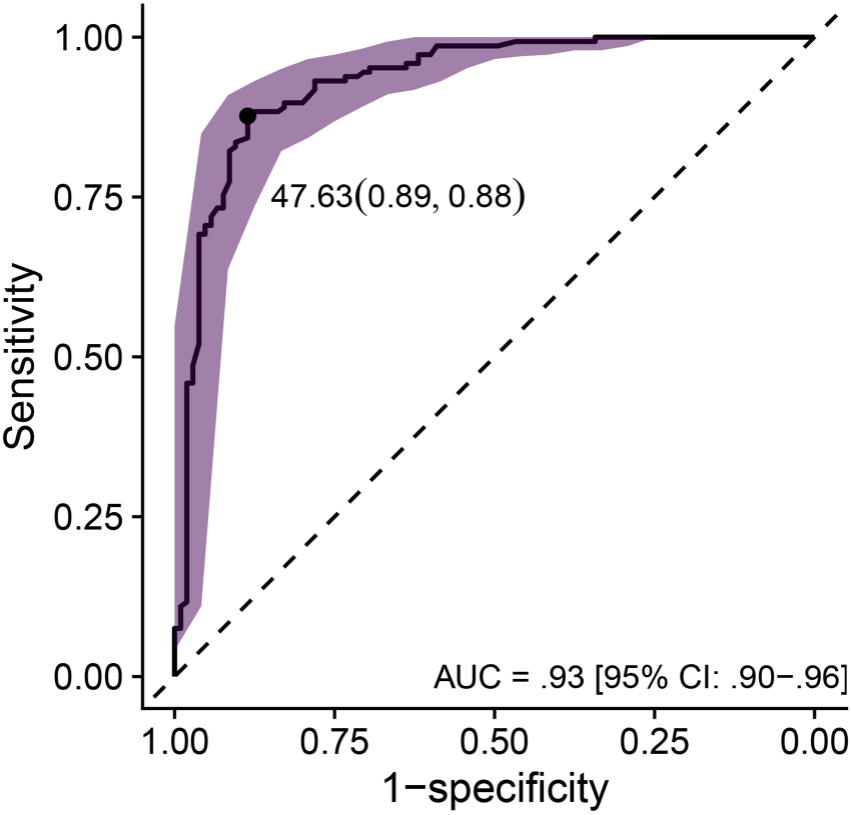
ROC curve visualizing the performance of the hearing test's binary classifier on the development dataset by plotting sensitivity as a function of 1 − specificity. The binary classifier was evaluated against the mild-HL referral criterion of a PTA_5_ greater than 35 dB HL in at least one ear and used the average of the estimated hearing thresholds at 4 and 6 kHz in both ears as the summary statistic. The ribbon represents the CI around the ROC.

**Figure 4.**
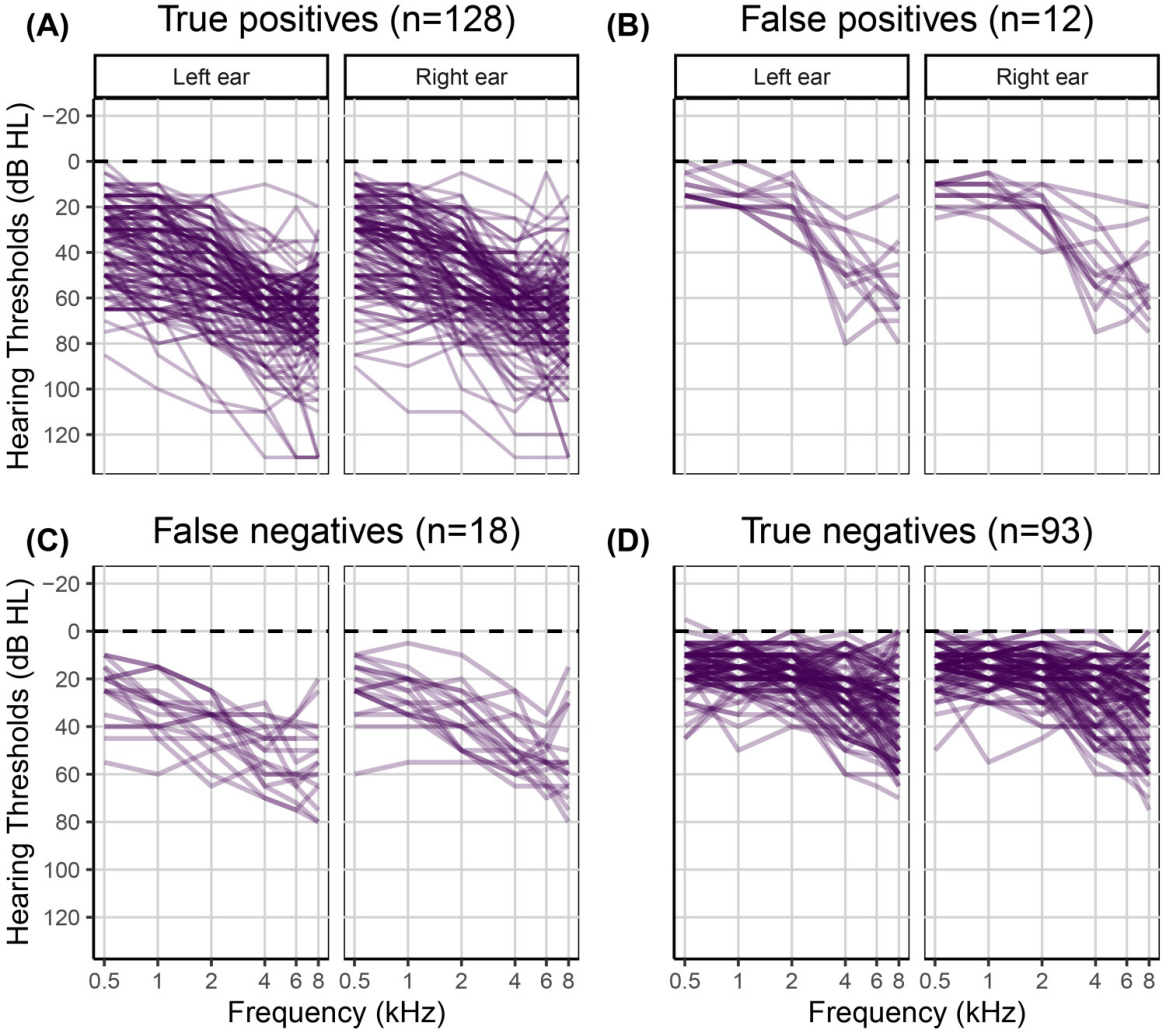
Individual clinical audiograms of the 251 participants in the development dataset. The left panels A and C show clinical audiograms for individuals with HL who were either correctly (true positives) or incorrectly (false negatives) classified by the hearing test. The right panels B and D show clinical audiograms for NH individuals who were either incorrectly (false positives) or correctly (true negatives) classified by the hearing test.

ROC curves plot sensitivity as a function of 1 − specificity (see Figure 3). Here, sensitivity referred to the proportion of participants who met the mild-HL criterion (*n* = 146 in the development dataset), who were correctly identified by the hearing test as having a HL. Sensitivity was calculated as the number of true positives divided by the sum of true positives and false negatives (Trevethan, 2017). Specificity referred to the proportion of participants with NH (*n* = 105 in the development dataset), who were correctly identified by the hearing test as having NH. Specificity was calculated as the number of true negatives divided by the sum of true negatives and false positives (Trevethan, 2017). As shown in Figure 3, sensitivity and specificity represent a trade-off and vary along a continuum, i.e., they vary with the pass/fail cutoff point. We optimized this cutoff point by maximizing the Youden index Jwith J=sensitivity+specificity−1 (Youden, 1950). Along with the cutoff point, we also needed to identify the optimal summary statistic of the estimated hearing thresholds. To this end, we explored arithmetic means across various subsets of the estimated hearing thresholds as the choice for the summary statistic, i.e., all 2- (*n* = 45), 4- (*n* = 210), 6- (*n* = 210), 8- (*n* = 45), and 10-frequency (*n* = 1) means. We computed the corresponding ROC curves and selected the summary statistic that yielded the largest Youden index overall. This was the arithmetic mean of the four estimated hearing thresholds at 4 and 6 kHz in both ears. It resulted in a Youden index J of 0.76, a cutoff point of 47.63 dB HL, an area under the curve of 0.93 [CI: 0.90, 0.96]), a sensitivity of 0.88 [CI: 0.82, 0.92], and a specificity of 0.89 [CI: 0.82, 0.94] for referring individuals in the development dataset with a clinical PTA_5_ greater than 35 dB HL in at least one ear. See Figure 3 for the ROC curve and Figure 4 for the clinical audiograms corresponding to the 128 true positives, 12 false positives, 18 false negatives, and 93 true negatives, respectively.

Sensitivity and specificity describe intrinsic characteristics of a test in reference to a gold standard diagnostic. Complementary to these, positive and negative predictive values are metrics describing the implications for an individual who obtains a certain test result. The positive predictive value (PPV) expresses the probability of someone truly having a condition if they are referred by a screening test (Trevethan, 2017). It is calculated as the number of true positives divided by the sum of true and false positives. The negative predictive value (NPV) states the probability of someone truly not having a condition if they pass the screening test (Trevethan, 2017). It is calculated as the number of true negatives divided by the sum of true and false negatives. Based on the development dataset, the PPV of the hearing test was 0.91 [CI: 0.87, 0.96], and the NPV was 0.84 [CI: 0.78, 0.90].

### Validation Dataset

Validation data were collected prospectively in Belgium between July and October of 2023. The participants constituted a convenience sample. They were considered for inclusion if they were 18 years of age or older. Real-world usage data suggest that online hearing tests are reaching a young demographic, with 45% of users under the age of 30 according to World Health Organization data (De Sousa et al., 2022) and 40% of users under the age of 40 according to usage data from this online hearing test (see Figure S1 in the Supplemental material). For the purpose of validating the hearing test, we aimed to recruit a sample representative of this user population. No requirements related to hearing status (NH vs. HL) or degree of HL were put forward. Individuals were contacted via e-mail or telephone. Those who were interested in taking part were visited at home, where they gave written informed consent prior to their participation in the study. All participants received a small financial compensation for their participation.

An initial sample of 159 participants was recruited. Three participants were excluded, as they did not consent to share their data outside of the University Hospitals or the European Union, resulting in a final sample of *n* = 156. Three student hearing care providers (HCPs) visited the participants in their homes, where all of them performed the hearing test using one of three hardware set-ups: a Dell Latitude13 7320 Detachable tablet (*n* = 69), a Lenovo IdeaPad c340-14ILW Windows laptop (*n* = 51), or a Microsoft Surface tablet (*n* = 36), combined with Sennheiser HD 300 headphones. The hardware set-ups were not calibrated and reflected real-world equipment that individuals might use at home. Moreover, Sennheiser HD 300 headphones were not among the transducers that were included in the transducer calibration measurements during the development of the hearing test. Participants performed the hearing test independently, relying solely on the instructions displayed on the screen. They did not receive instructions or assistance from the student HCPs. The hearing test was administered in the same way as described in the section Development Dataset, with the exception that the participants were instructed to set the volumes of the tablets and laptops at 100% (instead of 50%, as described in the section Audiogram Estimation). The participants and student HCPs were blinded to the outcomes of the hearing test. Following the hearing test, i.e., in the same session, the student HCPs measured clinical pure-tone air and bone conduction audiograms for the participants in their homes. All octave frequencies between and including 0.25 and 8 kHz were tested, as well as 6 kHz. The student HCPs employed the Hughson–Westlake 5-up-10-down method (Carhart & Jerger, 1959) using portable Madsen 662 audiometers, RadioEar DD65 headphones with Peltor caps, and RadioEar B71 bone conductors. Nüsse et al. (2014) previously had shown that reliable audiometric results could be obtained in home environments using a similar set-up.

## Results

**Figure 5.**
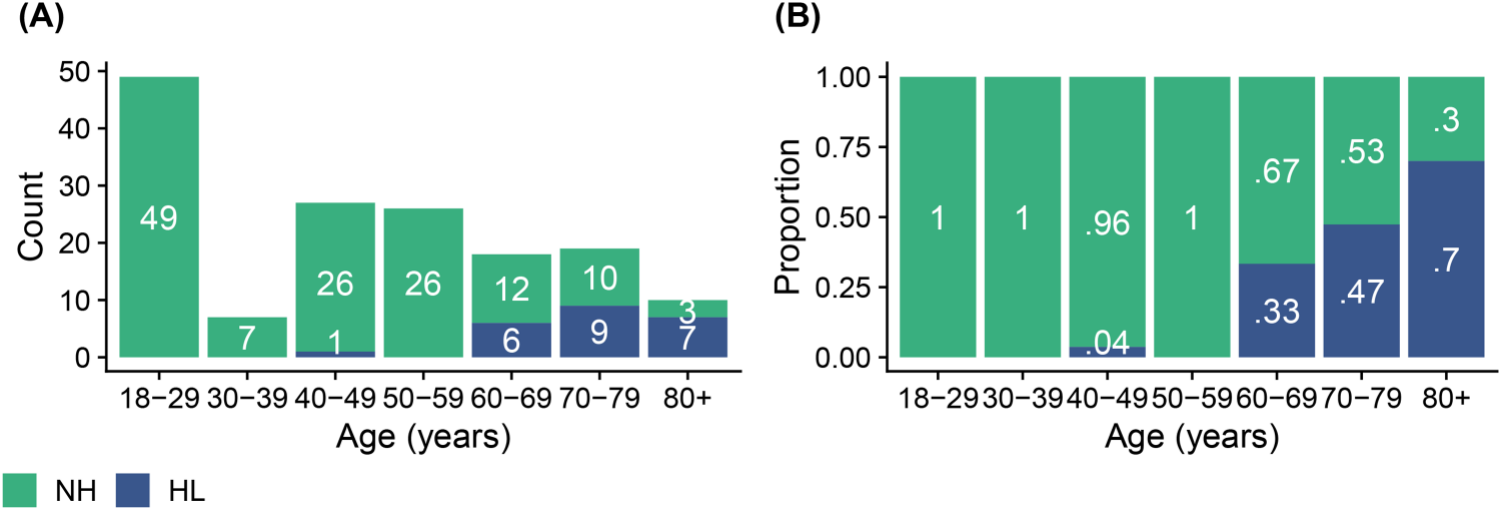
The number (panel A) and proportion (panel B) of participants in the validation dataset (*n* = 156), as a function of their age decade. Hearing status (NH versus HL) was determined per the mild-HL criterion.

Figure 5 visualizes the number of participants recruited for the validation of the hearing test per their age decade. It also shows their hearing status based on the mild-HL criterion of a clinical PTA_5_ greater than 35 dB HL in at least one ear. According to this mild-HL criterion, the validation dataset included 133 participants (85%) without and 23 participants (15%) with HL. Five of the 156 participants had air-bone gaps exceeding 15 dB at multiple frequencies, but no one was excluded because of air-bone gaps. There were no missing values in the validation dataset and no adverse events were reported during data collection.

### Hearing Screening Outcomes

**Figure 6.**
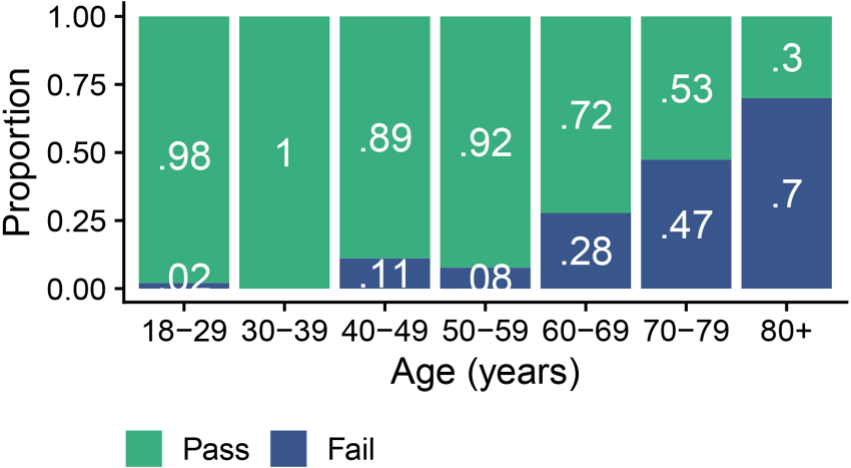
The proportion of pass and fail hearing test outcomes as a function of age for the 156 participants in the validation dataset.

****The primary aim of the hearing test was to screen for HL, that is, to provide a reliable pass/fail result. All 156 participants completed the hearing test. Figure 6 visualizes the hearing test outcomes as a function of age for the validation dataset. Across all ages, the algorithm classified 129 participants (83%) as passing and 27 participants (17%) as failing the hearing test.

**Figure 7.**
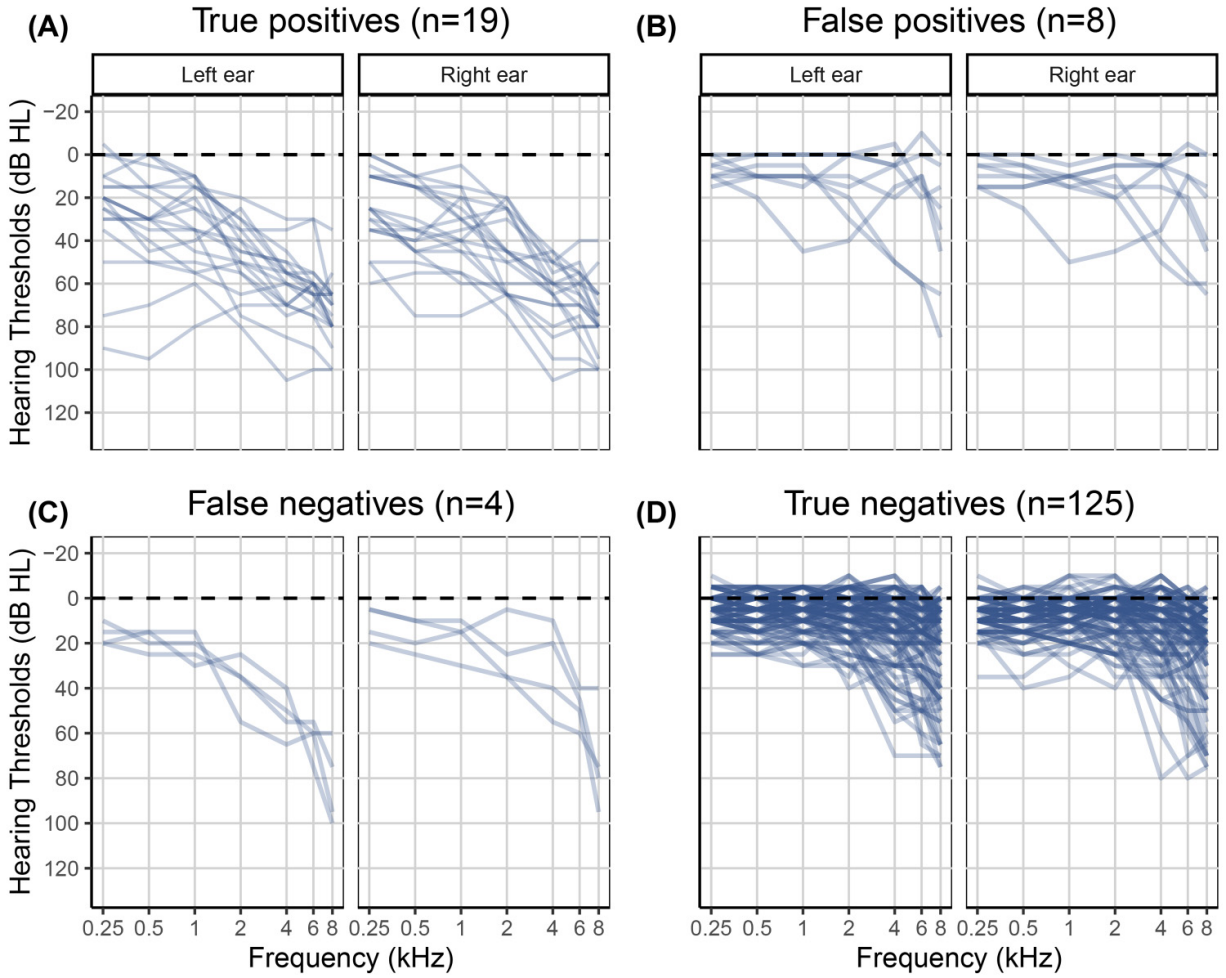
Clinical audiograms of the 156 participants in the validation dataset. The left panels A and C show clinical audiograms for participants with a HL who were either correctly (true positives) or incorrectly (false negatives) classified by the hearing test. The right panels B and D show clinical audiograms for NH participants who were either incorrectly (false positives) or correctly (true negatives) classified by the hearing test.

The classification outcomes were evaluated against the mild-HL criterion, that is, a clinical PTA_5_ greater than 35 dB HL in at least one ear. This analysis revealed 19 true positives, eight false positives, four false negatives, and 125 true negatives. Figure 7 shows the gold standard clinical audiograms for each of the four groups. In summary, based on the validation dataset and using the PTA_5_ criterion, the screener performed with a sensitivity of 0.83 [CI: 0.65, 0.96] and specificity of 0.94 [CI: 0.89, 0.98]. The PPV was 0.70 [CI: 0.57, 0.86], and the NPV was 0.97 [CI: 0.94, 0.99].

### Audiogram Estimation Outcomes

The secondary aim of the hearing test was to estimate a full bilateral audiogram. Outcome metrics for the validation of the estimated audiograms were the mean difference between estimated and gold standard hearing thresholds as well as the standard deviation of the differences, similar to the Bland–Altman method for judging agreement between two measurement methods (Bland & Altman, 1986, 1999). Differences were calculated as estimated minus gold standard clinical hearing thresholds for each participant, ear, and frequency. Visual inspection of the difference distributions did not reveal deviations from normality.

**Figure 8.**
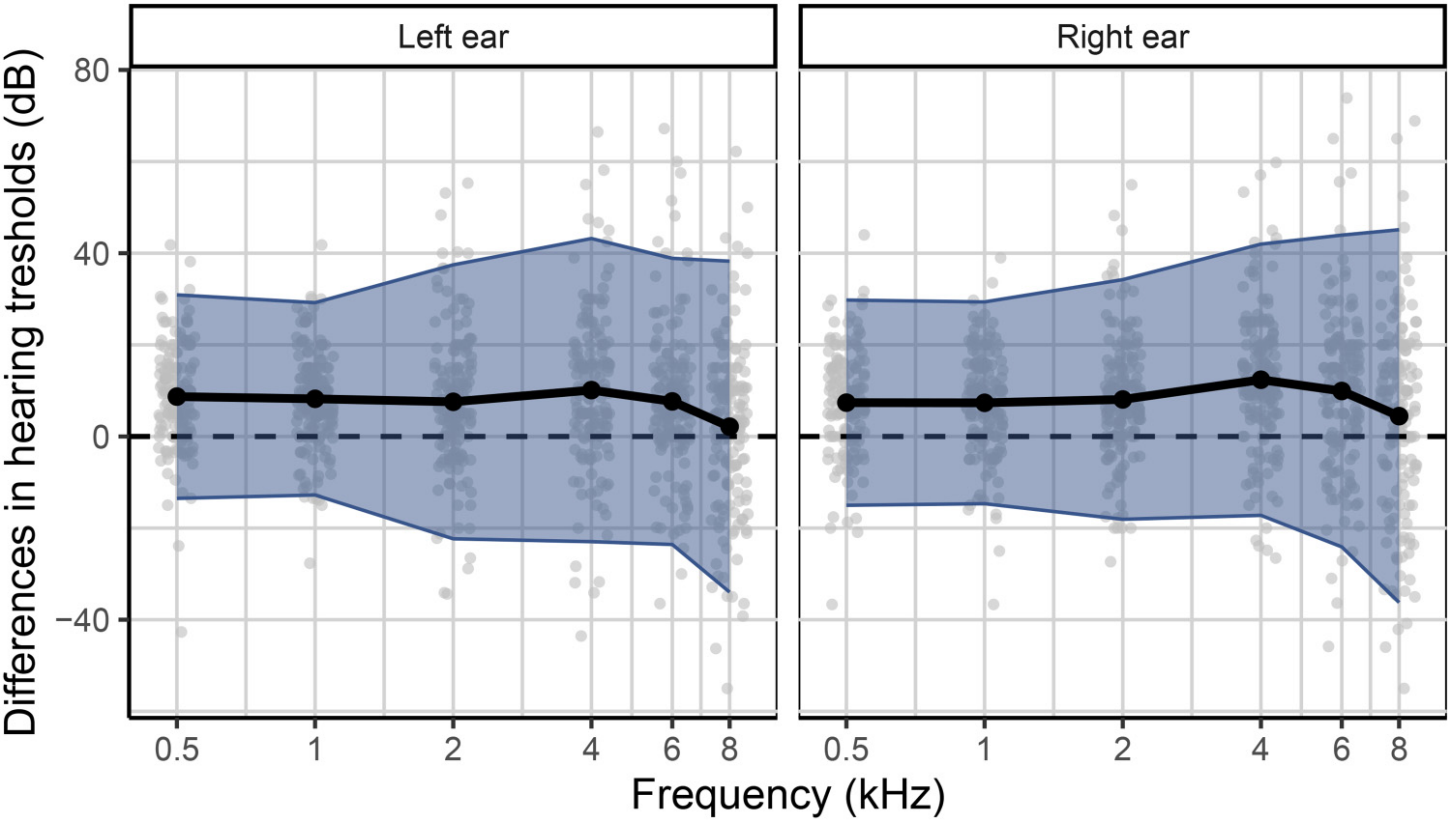
Scatter plot of differences between estimated and gold standard hearing thresholds as a function of frequency for the validation dataset (*n* = 156). Bullets represent individual data points, that is, 156 values per frequency. The solid curve shows the mean difference as a function of frequency. The ribbon represents the CIs for the individual differences (±1.96 SD).

Mean differences, that is, the individual differences averaged across the 156 participants in the validation sample, varied between 2.1 and 12.4 dB depending on ear and frequency (see Table 2 and Figure 8). The average standard deviation across ear and frequency was 14.8 dB. Table S2 in the Supplemental material lists the mean differences for each of the four hearing screener classification outcomes separately, that is, for the 19 true positives, eight false positives, four false negatives, and 125 true negatives.

**Table 2.** Mean Differences, that is, Individual Differences Between Estimated and Gold Standard Hearing Thresholds in dB Averaged Across the 156 Participants in the Validation Sample, Along with Standard Deviations in Parentheses.

	0.5 kHz	1 kHz	2 kHz	4 kHz	6 kHz	8 kHz
Left ear (*n* = 156)	8.7 (11.3)	8.2 (10.7)	7.6 (15.3)	10.1 (16.9)	7.7 (15.9)	2.1 (18.4)
Right ear (*n* = 156)	7.4 (11.4)	7.3 (11.2)	8.1 (13.3)	12.4 (15.1)	9.9 (17.4)	4.4 (20.8)

## Discussion

The COVID-19 pandemic intensified the need for a self-administered hearing test that would reliably screen for HL and could give an estimate of an individual's audiogram. To this end, we developed and validated the self-administered online hearing test described here. The test is easy and efficient to use, relying only on an internet connection, an internet browser such as running on a laptop, tablet, or smartphone, and headphones or earphones. During the test, individuals are asked to indicate their age decade, respond to three questions about their hearing abilities (Q_2_, Q_4_, and Q_5_ in Table 1), and self-assess four hearing thresholds following on-screen instructions.

We investigated the performance of the hearing test in an external validation study conducted in Belgium with 156 adult participants who had not taken part in the development study. The sample reflected a predominantly younger population, similar to those reached by online hearing screeners (cf. De Sousa et al., 2022, and Figure S1). We found that the newly developed hearing test screened for mild clinical HL or greater (defined as a PTA_5 _> 35 dB HL) with a sensitivity of 0.83 and specificity of 0.94. The hearing test thus correctly classified 83% of validation-study participants with a HL and 94% of participants with NH. In order to facilitate comparison with previous studies that employed a PTA_4_ definition of mild HL (see Van den Borre et al., 2021, for a review), we also calculated the screening outcome metrics for the scenario in which a mild clinical HL or greater was defined as a PTA_4 _> 31.3 dB HL (representing the PTA_4_ for Bisgaard et al., 2010, mild-HL standard N2 audiogram). This resulted in a sensitivity of 0.86 and a specificity of 0.93. The similarity of these values with those stated above indicates that sensitivity and specificity were not highly dependent on whether a PTA_4_ or PTA_5_ was used to define HL. The screening performance was comparable with that of the widely used digits-in-noise test. Depending on test sample demographics, language of test stimuli, and type of masking noise used, sensitivity values for the digits-in-noise test ranged between 0.73 and 0.94, and specificity values ranged between 0.65 and 0.95 when screening for mild HL (see Table 1 in Van den Borre et al., 2021). In comparison, Feltner et al. (2021) reviewed ten studies investigating single self-report questions to screen for mild HL in adults over 50 years of age and found a pooled sensitivity of 0.66 and specificity of 0.76.

Sensitivity and specificity values describe intrinsic performance characteristics of a test in reference to a gold standard. PPVs and NPVs, in turn, facilitate clinical interpretation of an individual test result. As PPVs and NPVs depend on prevalence, they ought to be determined for a sample representative of the intended user population in order to be valid for the real-world application (Power et al., 2013). In the case of an online hearing test, such a sample would consist of more individuals with NH than with HL, as was the case for our validation dataset. The validation data showed a probability of 0.70 for individuals who failed the hearing test to have a HL (PPV). The probability for individuals who passed the hearing test to have NH was 0.97 (NPV). There are few reports in the literature on the PPVs and NPVs of hearing screening methods. Potgieter et al. (2018) observed a PPV of 0.90 and NPV of 0.86 for the South African English smartphone digits-in-noise test, when screening for mild HL defined in terms of the PTA_4_. These values were determined for a balanced sample in which a little over 50% of study participants had a HL. Due to their dependence on HL prevalence, PPVs and NPVs would likely be markedly different in a representative sample with fewer positive (HL) cases (cf. Figure 9-6 in Mausner & Kramer, 1985). This is consistent with other studies that reported PPVs and NPVs in evaluating self-report measures to screen for HL: Screening for mild HL based on PTA_4_ in unbalanced samples yielded PPVs between 0.61 and 0.86 and NPVs between 0.43 and 0.82 (Clark et al., 1991; Sindhusake et al., 2001; Valete-Rosalino & Rozenfeld, 2005).

Note that NPVs would ideally be high when screening for HL, as in this study. High NPVs correspond to few false negatives (Trevethan, 2017), indicating that few individuals with HL are missed by a test. This is important in light of the burden that comes with unaddressed HL (Livingston et al., 2024; McDaid et al., 2021; World Health Organization, 2021b). In turn, medium-to-high PPVs are acceptable when screening for HL. A lower PPV implies more false positives, suggesting that more individuals with NH are referred for further testing, which is acceptable given that follow-up testing for HL is not harmful, stressful, or expensive for most individuals (Trevethan, 2017). According to our primary outcome metrics for hearing screening, the results of the validation study confirmed that the present hearing test is effective in screening for mild clinical HL.

In addition to serving as a hearing screener, a secondary purpose of the hearing test was to provide an estimated audiogram. Measuring audiograms remotely using self-administered hearing tests is challenging, given the presence of ambient noise in home environments, the use of uncalibrated equipment, and the potential for usage errors when tests are performed without supervision. Therefore, we developed a predictive algorithm to estimate the audiogram and evaluated it in terms of the differences between estimated and gold standard hearing thresholds. The algorithm was optimized based on the development dataset (*n* = 251). For that dataset, the mean differences between estimated and gold standard hearing thresholds ranged from −1.7 to 2.6 dB, and the average standard deviation of the differences was 15.0 dB. For the validation dataset (*n* = 156), mean differences were greater. They ranged from 2.1 to 12.4 dB with a mean of 7.8 dB. However, the average standard deviation of 14.8 dB was comparable with that for the development data. The mean differences averaged across all participants in the validation study provide only partial insight. The patterns of differences exhibited notable variation across the four hearing screening classification outcomes (see Table S2). Mean differences were smallest for the 19 true positives, ranging from −12.0 to 1.7 dB with a mean of −3.1 dB (Figure 7, panel A). These individuals, who were correctly classified by the hearing test as having HL, would receive a recommendation for follow-up with a clinician. The mean differences indicated greater bias, on average 8.8 dB, for the 125 true negatives, that is, for those individuals who were correctly classified by the hearing test as having NH (Figure 7, panel D). A high prevalence of young NH participants was responsible for the greater mean differences in this group. About one third of them showed negative hearing thresholds, i.e., clinical hearing thresholds better than 0 dB HL. The development dataset, in contrast, did not contain such audiograms (Figure 4) due to the high prevalence of middle-aged and older participants in that sample (Figure 2). Consequently, the regression stage of the predictive algorithm extrapolated the medial thresholds for the young NH participants in the validation study and produced larger estimation errors. Mean differences were also larger for the eight false positives and showed some left/right-ear asymmetries for the four false negatives (see Table S2). These individuals were misclassified by the hearing test. This was, at least in part, attributable to their gold standard audiograms: One false positive showed a cookie bite HL, two false positives showed steeply sloping HLs, and three of the four false negatives had asymmetric HLs (Figure 7, panels B and C). These audiometric shapes are less common in the population at large and therefore would have found few close matches among the prototype audiograms. Additionally, such matches would have been heavily penalized in terms of low Bayesian priors. Furthermore, some audiogram estimation errors can be attributed to misreported age and discrepancies between self-reported hearing difficulties and audiometric thresholds. For example, one of the false positives incorrectly entered their age as 70–79 years while their true age was 18–29 years. They had a normal clinical audiogram, with all thresholds falling within the range of −5 to 5 dB HL. Yet they rated their hearing as poor and indicated that they often had difficulty on the telephone and with hearing high-pitched sounds. The integration of self-report and measured thresholds in the predictive algorithm reaches its limits when the perceived hearing abilities differ from what would generally be suggested by the clinical audiogram. The algorithm aims to strike an optimal balance between robust estimation and the consideration of rare individual cases. Three of the eight false positives rated their hearing as poor, two indicated they were unsure, and another often had difficulty hearing high-pitched sounds. In other words, 75% of the false positives perceived some degree of hearing difficulty. Thus, the recommendation to see a clinician for follow-up would not have been unwarranted for them, as they might benefit from interventions such as aural rehabilitation and counseling. In general, we consider the audiogram estimation performance, the secondary purpose of the hearing test, to be acceptable, given that the estimated audiograms for true positives, i.e., those who would actually be referred for follow-up testing, exhibited minimal bias and that false positives and false negatives were relatively rare (5% and 3% of individuals, respectively, in the validation dataset). Nevertheless, the average standard deviation for the true positives was 14.3 dB, indicating relatively strong fluctuations around the mean difference.

We would have liked to compare the audiogram estimation performance of this hearing test with that of existing tests. Unfortunately, we are not aware of any other self-administered online or mobile applications that provide audiogram estimates and have been externally validated in real-world home environments using a variety of uncalibrated equipment. Oremule et al. (2024) reviewed 17 studies investigating the accuracy of mobile audiometric applications. However, only one of the reviewed studies with adult participants, Renda et al. (2016), appeared to meet all of the following criteria with regard to evaluation: self-administered testing, that is, without facilitation or assistance by professionals, outside of a soundproof booth, and not calibrated exclusively to a single headphone or earphone model. Renda et al. (2016) had 100 individuals perform the Hearing Test™ (e-audiologia.pl) in a quiet clinic room on a Samsung Galaxy S4 smartphone with the headphones that came with the phone. In contrast to our validation study, their participants were pre-screened for normal tympanometric results and their ability to perform the test. Renda et al. observed mean differences ranging from −3.6 to 4.6 dB (as derived from their Table 2). This indicates a somewhat smaller bias than that observed for our hearing test in the validation study (see Table 2). Unfortunately, Renda et al. did not report the standard deviation of the signed differences, precluding a comparison of the precision of the audiogram estimated by the Hearing Test™ and our hearing test. More recently, Moazzami et al. (2024) evaluated the Mimi Hearing Test iOS smartphone application (Mimi Hearing Technologies GmbH) in a balanced sample of 75 adults (51% of the ears were NH). In contrast to our validation study, these participants were pre-screened for the absence of cognitive impairment, otorrhea, and earwax impaction. The test was conducted on an iPhone X in a quiet room at an audiology clinic, utilizing either Sennheiser HDA200 or HDA300 audiometric headphones. Testing was self-administered but assisted: “Before completing the mobile evaluation, participants were briefly instructed on how to use the application by a member of the research team” (Moazzami et al., 2024). The test took an average of 10 minutes. Sixteen percent of the 75 participants received inconclusive or incomplete test results in one ear, and another 16% in both ears. In contrast, all participants in our validation study successfully completed the hearing test. Moazzami et al. observed mean differences at 0.5, 1, 2, 4, 6, and 8 kHz of 21.1, 11.7, 9.4, 6.4, 2.5, and 0 dB, respectively, for the HDA200 headphones and 17.4, 17.8, 14.8, 5.0, 6.3, and 6.3 dB, respectively, for the HDA300 headphones. Thus, in comparison with our validation study, Moazzami et al. observed larger mean differences at low frequencies and a greater frequency dependence of these differences. The standard deviation of the signed differences was not reported, precluding a comparison with the standard deviation observed in our validation study. To the best of our knowledge, the current study is the first to validate a threshold hearing test at home, in uncontrolled environments, with various types of uncalibrated equipment, without pre-screening, and without the assistance of trained professionals. It would be expected that estimates obtained under such real-world conditions would be less precise than those obtained in clinic environments utilizing single headphones. Consequently, we anticipate that existing online or mobile applications would show standard deviations of differences similar to those observed for this test if they were subjected to realistic testing conditions.

As mentioned in the Introduction, having access to an estimated audiogram in the absence of a clinical audiogram would be beneficial from a clinical point of view. For example, it provides insight into the severity of the HL and could facilitate early counseling, even before a thorough diagnostic assessment was available. In the context of remote hearing care, an estimated audiogram could potentially be used for pre-fitting adjustments of hearing aids prior to delivery to the patient, after which in-situ audiometry should be performed (Van Eeckhoutte et al., 2024; Vercammen, 2020). Non-specialist personnel such as community health workers could be trained to perform such procedures in low-resource settings (Dillard et al., 2024; World Health Organization, 2023). However, the use of the estimated audiogram as part of remote care would require consideration of other aspects of hearing rehabilitation beyond the scope of this study. These include considerations pertaining to the inspection of the external ear and otoscopy, clinical diagnosis, advanced auditory and non-auditory diagnostics, HL etiology, differential diagnosis, the selection of aural rehabilitation strategy and assistive technology, the provision of counseling, and integrated care (Boothroyd, 2007; Roeser et al., 2007; Saunders et al., 2021). Even when considering the audiogram alone, it is important to note that the hearing test described here was not intended to be, and cannot be, a replacement for gold standard clinical audiometry. Its principal aim was to reliably screen for hearing loss, and this external validation study demonstrated that it achieves this objective.

Finally, in addition to developing a robust hearing screener, we aimed to develop an efficient one. In contrast to existing online hearing screeners, we chose to use a Bayesian approach leveraging probabilistic effects of age as well as interfrequency and interaural relationships in human audiograms. Therefore, the test required hearing threshold determination at four frequencies only, two in the right and two in the left ear. Online usage data from more than 100,000 website visitors (see Figure S1) confirmed the efficiency of this test: The median testing time was 2.8 min with an interquartile range of 1.9 min.

## Conclusion

We described the development and validation of a self-administered online hearing test for use in hearing screening. In addition to providing a screening outcome, the test also generates an estimated audiogram. In contrast to existing online hearing screeners, this test is based on a predictive algorithm that combines self-report with pure-tone threshold measures through multiple regression to reduce the test's susceptibility to measurement uncertainty and error. The predictive algorithm further encompasses a Bayesian classifier that leverages probabilistic effects of age, interfrequency and interaural relationships in human audiograms, and a binary classifier that produces a pass/fail result. The test can be completed online, at home, is efficient, requires minimal equipment, and no prior experience. The external validation results demonstrated that its screening performance is comparable to that of other state-of-the-art hearing screeners. With respect to the secondary outcome of audiogram estimation, no other method has been evaluated under similarly realistic conditions, thereby rendering performance comparisons difficult. In summary, this test provides a validated alternative to available screeners, and, additionally, it provides an estimated audiogram.

## Supplemental Material

sj-docx-1-tia-10.1177_23312165251317923 - Supplemental material for Development and Validation of a Self-Administered Online Hearing Test Supplemental material, sj-docx-1-tia-10.1177_23312165251317923 for Development and Validation of a Self-Administered Online Hearing Test by Charlotte Vercammen and Olaf Strelcyk in Trends in Hearing
