# Supplementary material for: Development and Validation of a Self-Administered Online Hearing Test
Source: Trends Hear. 2025 Mar 18;29:23312165251317923. doi: 10.1177/23312165251317923 (PMC11920986; doi:10.1177/23312165251317923)
Supplement: sj-docx-1-tia-10.1177_23312165251317923 - Supplemental material for Development and Validation of a Self-Administered Online Hearing Test [file sj-docx-1-tia-10.1177_23312165251317923.docx]

**Supplementary material**


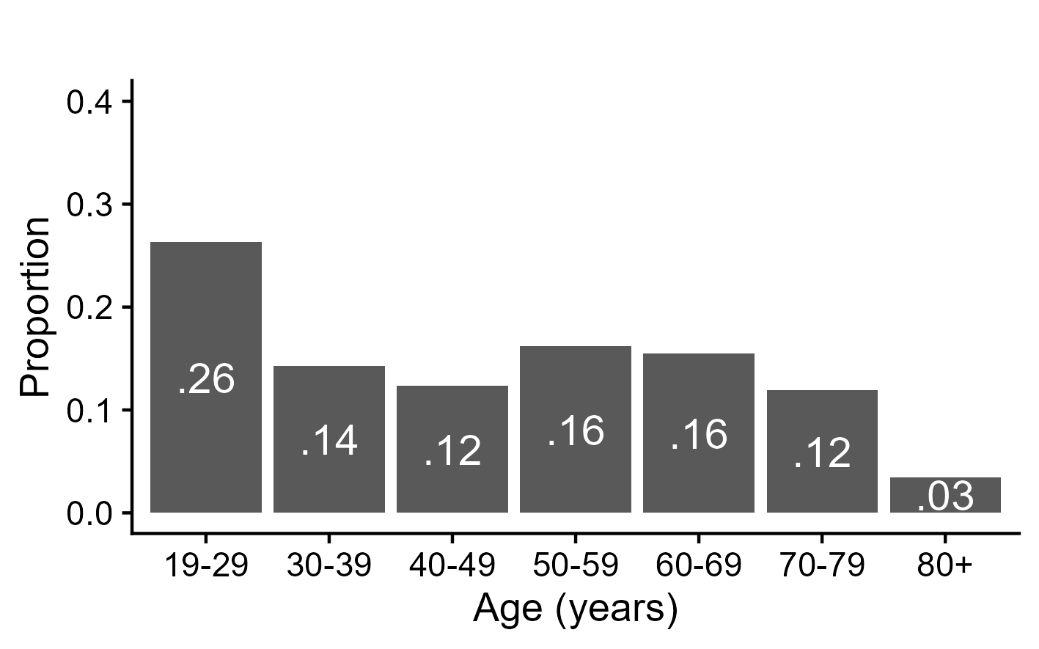


*Figure S1. Age distribution of 100,419 organic website visitors who completed the online self-administered hearing test. 40% were under the age of 40 years. Online usage data from the same users showed that the median testing time was 2.8 minutes (interquartile range: 1.9 minutes).*

*Table S1. STARD checklist from Bossuyt et al. (2015), as can be downloaded from* [*www.equator-network.org/reporting-guidelines/stard*](http://www.equator-network.org/reporting-guidelines/stard)*, and applied to the external validation of the hearing test.*

|  | **Section & Topic** | **No** | **Item** | **Reported on page #** |
| --- | --- | --- | --- | --- |
|  |  |  |  |  |
|  | **TITLE OR ABSTRACT** |  |  |  |
|  |  | **1** | Identification as a study of diagnostic accuracy using at least one measure of accuracy (such as sensitivity, specificity, predictive values, or AUC) | 3 |
|  | **ABSTRACT** |  |  |  |
|  |  | **2** | Structured summary of study design, methods, results, and conclusions (for specific guidance, see STARD for Abstracts) | 3 |
|  | **INTRODUCTION** |  |  |  |
|  |  | **3** | Scientific and clinical background, including the intended use and clinical role of the index test | 5 |
|  |  | **4** | Study objectives and hypotheses | 5 |
|  | **METHODS** |  |  |  |
|  | *Study design* | **5** | Whether data collection was planned before the index test and reference standard were performed (prospective study) or after (retrospective study) | 13 |
|  | *Participants* | **6** | Eligibility criteria | 13 |
|  |  | **7** | On what basis potentially eligible participants were identified  (such as symptoms, results from previous tests, inclusion in registry) | 13 |
|  |  | **8** | Where and when potentially eligible participants were identified (setting, location and dates) | 13 |
|  |  | **9** | Whether participants formed a consecutive, random or convenience series | 13 |
|  | *Test methods* | **10a** | Index test, in sufficient detail to allow replication | 7-13 |
|  |  | **10b** | Reference standard, in sufficient detail to allow replication | 11, 13-14 |
|  |  | **11** | Rationale for choosing the reference standard (if alternatives exist) | 11 |
|  |  | **12a** | Definition of and rationale for test positivity cut-offs or result categories of the index test, distinguishing pre-specified from exploratory | 11-13 |
|  |  | **12b** | Definition of and rationale for test positivity cut-offs or result categories of the reference standard, distinguishing pre-specified from exploratory | 11-12 |
|  |  | **13a** | Whether clinical information and reference standard results were available to the performers/readers of the index test | 13-14 |
|  |  | **13b** | Whether clinical information and index test results were available  to the assessors of the reference standard | 13-14 |
|  | *Analysis* | **14** | Methods for estimating or comparing measures of diagnostic accuracy | 12-13 |
|  |  | **15** | How indeterminate index test or reference standard results were handled | NA |
|  |  | **16** | How missing data on the index test and reference standard were handled | 14 |
|  |  | **17** | Any analyses of variability in diagnostic accuracy, distinguishing pre-specified from exploratory | 14 |
|  |  | **18** | Intended sample size and how it was determined | No power analysis performed |
|  | **RESULTS** |  |  |  |
|  | *Participants* | **19** | Flow of participants, using a diagram | Not included (convenience sample) |
|  |  | **20** | Baseline demographic and clinical characteristics of participants | 13-14 |
|  |  | **21a** | Distribution of severity of disease in those with the target condition | 14 |
|  |  | **21b** | Distribution of alternative diagnoses in those without the target condition | NA |
|  |  | **22** | Time interval and any clinical interventions between index test and reference standard | 13 |
|  | *Test results* | **23** | Cross tabulation of the index test results (or their distribution)  by the results of the reference standard | 14 |
|  |  | **24** | Estimates of diagnostic accuracy and their precision (such as 95% confidence intervals) | 14 |
|  |  | **25** | Any adverse events from performing the index test or the reference standard | 14 |
|  | **DISCUSSION** |  |  |  |
|  |  | **26** | Study limitations, including sources of potential bias, statistical uncertainty, and generalisability | 16-19 |
|  |  | **27** | Implications for practice, including the intended use and clinical role of the index test | 16-19 |
|  | **OTHER INFORMATION** |  |  |  |
|  |  | **28** | Registration number and name of registry | Not preregistered. |
|  |  | **29** | Where the full study protocol can be accessed | Upon request |
|  |  | **30** | Sources of funding and other support; role of funders | 2 |
|  |  |  |  |  |

*Table S2. Mean differences, i.e., individual differences between estimated and gold standard hearing thresholds in dB averaged across subgroups of participants in the validation sample, along with standard deviations in parentheses. The four subgroups corresponded to the four hearing screener outcome groups for the validation sample, i.e., true positives, false positives, false negatives, and true negatives.*

|  |  | 0.5 kHz | 1 kHz | 2 kHz | 4 kHz | 6 kHz | 8 kHz |  |
| --- | --- | --- | --- | --- | --- | --- | --- | --- |
| True Posit. (n=19) | Left | 1.7 (17.8) | 0.1 (14.5) | -2.4 (17.0) | -2.1 (15.3) | -1.2 (13.2) | -10.6 (11.2) |  |
|  | Right | 1.1 (17.1) | -2.1 (15.6) | -1.8 (14.0) | -4.4 (12.0) | -3.3 (10.4) | -12.0 (13.5) |  |
| False Posit. (n=8) | Left | 26.0 (10.1) | 22.3 (12.6) | 30.0 (22.1) | 36.8 (24.3) | 37.4 (31.4) | 23.7 (34.7) |  |
|  | Right | 23.3 (10.3) | 18.3 (15.4) | 30.0 (19.1) | 35.0 (24.4) | 37.9 (32.1) | 29.1 (33.5) |  |
| False Negat. (n=4) | Left | -2.5 (9.1) | -6.2 (8.9) | -17.5 (12.8) | -16.6 (19.3) | -17.4 (4.7) | -37.4 (16.6) |  |
|  | Right | -1.8 (13.2) | -3.1 (15.0) | -4.7 (16.3) | -0.6 (11.1) | -10.8 (12.0) | -34.6 (19.9) |  |
| True Negat. (n=125) | Left | 9.0 (8.7) | 9.0 (8.5) | 8.5 (12.2) | 11.1 (13.7) | 7.9 (12.1) | 4.0 (14.9) |  |
|  | Right | 7.6 (9.2) | 8.4 (8.8) | 8.6 (10.8) | 13.9 (12.0) | 10.8 (14.4) | 6.6 (17.5) |  |
